# Multisource Sensor Fusion and Large Language Model Integration for Explainable State Perception and Anomaly Awareness

**DOI:** 10.3390/s26154708

**Published:** 2026-07-24

**Authors:** Bocheng Zhou, Jinze Xie, Tiantian Chen, Bingyan Ning, Jingwen Cao, Yansong Dong, Manzhou Li

**Affiliations:** 1 China Agricultural University, Beijing 100083, China; 2 National School of Development, Peking University, Beijing 100871, China; 3 Beijing Forestry University, Beijing 100083, China

**Keywords:** multisource sensing signals, language–behavior consistency, large language models, explainable risk perception, intelligent monitoring systems

## Abstract

With the rapid development of intelligent sensing systems, digital monitoring platforms, and multisource data acquisition technologies, accurate identification of operational states and potential risks from heterogeneous sensing signals has become an important research issue in artificial intelligence-driven sensing. Existing studies have primarily focused on either textual information understanding or behavioral data analysis, with limited attention paid to jointly modeling the consistency between textual declarations and executed behaviors. As a result, many potential risks that have not yet manifested as significant anomalies but already involve execution deviations are difficult to detect in a timely manner. To address this issue, a language–behavior consistency sensing framework for multisource sensing signals is proposed. Textual sensing signals and behavioral sensing signals are mapped into a shared state logic space, and intelligent perception and quantitative analysis of deviations between textual states and executed states are achieved through a textual state logic extraction module, an observed behavioral state modeling module, and a language–behavior consistency measurement module. Systematic experiments were conducted on a multisource sensing dataset containing public declaration texts, operation reports, behavioral logs, resource allocation records, and state-response information. The results show that the proposed method achieved the best performance in the baseline comparison experiment, with a language–behavior consistency score (LCS) of 0.742, an AUC of 0.846, an F1-score of 0.811, a Precision of 0.802, and an explanation consistency score (ECS) of 0.821, clearly outperforming advanced methods such as FinBERT, LSTM, Multimodal Transformer, and the Contrastive Multimodal Model. These results demonstrate that language–behavior consistency sensing can effectively fuse multisource sensing information and improve complex system state identification, anomaly early warning, and risk perception, providing an interpretable artificial intelligence-driven sensing framework with the potential to support industrial operation and maintenance, intelligent manufacturing, digital infrastructure management, and other intelligent monitoring scenarios.

## 1. Introduction

With the rapid development of intelligent sensing systems, digital monitoring platforms, and multisource data acquisition technologies, identifying potential state deviations between external representations and actual operational behaviors has become an important issue in intelligent sensing, system diagnosis, and state perception [1]. In practical applications, industrial systems, platform systems, device clusters, operation-and-maintenance entities, and management units often express target states, operational constraints, and control strategies through operation reports, technical announcements, configuration files, inspection records, risk alerts, and meeting minutes [2]. These materials can be regarded as textual sensing signals that reflect textual states and operational intentions [3]. Meanwhile, device operation parameters, resource scheduling records, task execution intensity, load variations, and performance fluctuations constitute behavioral sensing signals that reflect actual execution states and dynamic responses [4]. Persistent inconsistency between textual and behavioral signals may indicate operational risks, control failures, or execution biases before observable anomalies emerge [5,6]. Therefore, developing an intelligent sensing framework capable of jointly understanding textual and behavioral sensing signals and identifying their logical consistency is of significant importance [7]. Traditional state assessment methods mainly focus on behavioral outcomes such as resource utilization, load levels, structural parameters, and performance variations, whereas target objectives, constraints, and control logic contained in textual declarations are rarely incorporated into a unified sensing framework [8]. However, textual data not only contain information content but also encode operational objectives, constraint conditions, and control preferences [9]. When behavioral sensing signals systematically deviate from these declarations, latent contradictions may exist between textual states and executed states [10]. Unlike conventional anomaly detection, language–behavior consistency sensing focuses on whether actual behaviors violate the constraints and operational logic expressed in textual signals, thereby providing a more forward-looking perspective for risk perception and state identification [11,12,13].

Existing studies have attempted to exploit textual information for state identification, risk perception, and intelligent decision-making [14]. Traditional approaches commonly rely on keyword dictionaries, semantic lexicons, or manually designed rules to quantify textual information and investigate its relationship with system responses and operational states [15]. Although these methods are interpretable, they often capture only surface-level semantics and struggle to model contextual dependencies and latent logical relationships in complex textual sensing signals [16,17,18]. Furthermore, textual information is frequently treated as a proxy for sentiment or information content, while the consistency between declared logic and subsequent behavioral execution remains largely unexplored [19,20]. In parallel, state identification and anomaly detection studies have traditionally relied on behavioral sensing signals, including operational indicators, sensor measurements, resource flows, and performance fluctuations [21,22]. Statistical models, time-series models, and machine learning approaches have demonstrated strong capability in detecting abnormal behavioral patterns [23]. Nevertheless, these methods generally assume that risks can be identified through statistical deviations in behavioral variables [24]. In practice, certain behaviors may remain statistically normal while simultaneously conflicting with previously declared operational intentions. Consequently, relying solely on behavioral sensing signals may overlook important declaration–execution deviations and fail to capture their underlying semantic implications [25,26,27]. Recent advances in deep learning, particularly pretrained language models and large language models, have provided new opportunities for textual sensing signal understanding [28]. Compared with dictionary-based approaches, these models can capture contextual semantics, implicit logic, and long-range dependencies, enabling structured extraction of operational objectives, constraints, preferences, and control strategies from complex textual materials [29,30,31]. However, most existing studies still employ textual information primarily for outcome prediction, while logical consistency between textual declarations and behavioral sensing signals remains underexplored [32,33]. Although recent research has investigated behavioral consistency, entity modeling, and multisource interaction mechanisms [34,35,36,37], a unified framework for language–behavior consistency sensing in multisource environments has yet to be established.

To bridge this gap, we propose a unified language–behavior consistency sensing framework that jointly models textual declarations and behavioral executions within a shared representation space. Textual materials are treated as sensing signals of textual states, while observable behavioral indicators are regarded as sensing signals of executed states. A large language model is employed to extract operational logic from textual sensing signals, a deep temporal network is used to model behavioral evolution, and a consistency measurement network is designed to quantify deviations between declaration logic and execution logic within a unified representation space. The main contributions of this study are summarized as follows.

1. A language–behavior consistency sensing framework is proposed to jointly model textual declarations and behavioral executions from a unified multisource sensing perspective.
2. A large-language-model-based logic extraction mechanism and a deep behavioral-state modeling mechanism are developed to generate comparable representations of textual states and executed states.
3. A language–behavior consistency indicator and consistency measurement network are designed to explicitly characterize declaration–execution deviations and improve state perception capability.
4. Extensive experiments demonstrate the effectiveness of the proposed framework for intelligent sensing, anomaly perception, and risk warning in complex systems.

To avoid ambiguity, the application domains discussed in the Introduction, including industrial operation and maintenance, intelligent manufacturing, digital infrastructure, and other representative scenarios, are intended as motivating examples illustrating the potential applicability of the proposed language–behavior consistency sensing framework rather than independent experimental domains. All experiments reported in this study were conducted on a unified multisource sensing dataset integrating textual sensing signals, behavioral sensing signals, and state-response information. The purpose of the experimental evaluation is to validate the general effectiveness and scalability of the proposed framework under a unified modeling setting instead of developing domain-specific solutions for individual application scenarios.

## 2. Related Work

### 2.1. The Role of Textual Sensing Signals in State Perception Research

In intelligent sensing and data-driven modeling research, textual data such as policy documents, corporate disclosure texts, news reports, announcements, and meeting minutes have gradually been regarded as important unstructured sensing signals. Unlike conventional numerical sensors, textual signals do not directly record physical or behavioral quantities, but reflect state descriptions, intention declarations, risk warnings, and external environmental changes through linguistic expression [14]. Such signals have diverse sources, large scale, high update frequency, and rich semantic information, thereby providing complementary information for complex system state identification, risk perception, and intelligent decision-making. Existing studies have generally treated text-derived indicators as proxy variables for uncertainty, sentiment, expectation changes, or state disturbances, rather than directly regarding text as a complete system operation mechanism [38]. In policy and public text perception research, news reports and public texts are often used to characterize external environmental uncertainty. The economic policy uncertainty index constructed by Baker et al. describes macro-level uncertainty states by counting the frequency of policy-related keywords in newspapers [39]. This type of method transforms large-scale text streams into computable time-series sensing indicators for reflecting potential disturbance intensity in complex environments. Hartley further indicated that large language models can significantly improve the measurement accuracy of such textual indicators and extend them to historical and multilingual corpora [40]. This indicates that textual sensing signals have gradually evolved from keyword statistics to contextual understanding and semantic classification tasks.

In organizational disclosure text perception research, annual reports, announcements, and earnings conference calls are often used to characterize information states, operational transparency, and future expectations. For example, the readability of financial reports is commonly regarded as a proxy for information complexity or opacity [38]. Loughran and McDonald noted that readability should be interpreted cautiously and is more suitable as a proxy for disclosure complexity or text processing difficulty [41]. Matera showed that narrative information in corporate earnings calls can improve the prediction of future earnings and analyst expectations [42]. Ignatov and Rudolf further found a positive correlation between forward-looking ESG disclosures and subsequent actual ESG performance, indicating that textual declarations may reflect subsequent behavioral trends [43]. In sentiment and public-opinion perception research, text analysis has been widely used to construct indicators of external environments or group responses. Classic studies have used news headlines and media wording to construct sentiment variables for explaining asset price fluctuations [44]. Domain-specific sentiment dictionaries and machine learning models have also been developed. García et al. constructed context-adapted positive and negative word lists based on stock price reactions, improving the predictive capability of textual sentiment indicators [45]. FinBERT, proposed by Huang et al., improves sentiment recognition in analyst reports through domain-specific pretraining [46]. Media sentiment and investor attention have also been shown to affect trading activities and risk premiums [47,48], and an investor sentiment index constructed from Chinese news data has predictive capability for extreme downside risk. Overall, textual sensing signals have been widely applied to policy communication, organizational disclosure, public-opinion monitoring, and state identification, and the extracted semantic features can serve as important complementary variables for complex system perception [14].

### 2.2. Behavioral Sensing Signals and Abnormal State Identification Research

Unlike textual sensing signals, behavioral sensing signals more directly reflect the execution state and dynamic response of a target entity in real-world environments. Behavioral data usually originate from continuous observations, transaction records, operation logs, indicator sequences, interaction behaviors, and structured state variables, and their core value lies in capturing pattern changes, abnormal fluctuations, and potential risks during system operation. For example, in the fields of fraud identification and abnormal transaction detection, Chen et al. systematically reviewed recent deep-learning-based fraud identification methods and presented the latest advances in using transaction flows and account networks to identify abnormal behaviors [49]. Overall, anomaly identification research centered on behavioral sensing signals has gradually formed a relatively mature paradigm and has become an important tool for identifying abnormal states in intelligent regulation, risk perception, and security monitoring [46]. However, existing behavior-data-based risk management models usually focus more on the statistical deviation or pattern mutation of behavioral signals themselves, while less attention is paid to whether behavioral states remain consistent with previous textual declarations of the entity. In other words, traditional behavioral anomaly detection methods are good at answering whether behavior is abnormal, but they do not necessarily answer whether behavior matches the textual state. In some scenarios, behavioral signals themselves may not yet exhibit extreme anomalies, but they may have already deviated from the objectives, constraints, or risk attitudes publicly expressed by the entity; such deviations may themselves constitute important early risk signals. Due to the lack of integration with textual signals, traditional risk models often have difficulty identifying such implicit inconsistency phenomena. In recent years, a few studies have begun to focus on the matching relationship between declarations and execution. For example, Abis and Lines found that when the actual holdings of mutual funds deviate from their declared strategies, fund inflows into comparable funds decline significantly [50]. This suggests that external observers regard the consistency between declarations and behaviors as an important basis for trust evaluation and risk judgment.

### 2.3. Research Gap in Language–Behavior Consistency Sensing

Textual sensing signal analysis and behavioral sensing signal modeling have achieved significant progress in state identification and risk perception, respectively, yet systematic quantitative research on language–behavior consistency remains relatively insufficient. Existing text-based methods mostly focus on sentiment, topics, readability, or information content, whereas existing behavioral methods mainly focus on statistical anomalies, sequence mutations, and pattern deviations; these two lines of research have long belonged to different modeling paradigms. The study by Abis and Lines on mutual funds showed that external participants penalize managers who fail to fulfill declared strategies [50]. Another example is the comparison between ESG narratives and actual ESG performance conducted by Ignatov and Rudolf, in which overall consistency was found between the two, but an “over-commitment” phenomenon was observed in certain high-pollution industries, where firms with poorer environmental performance emphasized environmental topics more strongly in reports [43]. These findings indicate that language–behavior inconsistency itself deserves to be regarded as a perceivable state-deviation signal; however, related studies remain limited to small-scale validation in specific contexts and have not yet developed into a scalable general modeling framework. An important reason for this research gap lies in the evident differences between textual data and behavioral data in data form, sampling frequency, semantic hierarchy, and modeling objectives. Textual signals are usually unstructured, semantically ambiguous, and strongly dependent on context, whereas behavioral signals are structured, temporal, and numerically fluctuating. How to map the two into a unified representation space and measure the matching degree between textual states and executed states constitutes a key challenge in multisource sensing signal fusion. With the development of natural language processing, large language models, and multimodal learning technologies, quantitative comparison of language and behavior consistency has gradually become feasible, thereby providing technical conditions for bridging this gap. Recent advances in cross-view and cross-modal representation learning have further improved the capability of modeling heterogeneous information. Representative methods such as CrossFormer employ hierarchical cross-view interaction mechanisms to enhance feature fusion across multiple views [51], while BiMAC introduces bidirectional multimodal alignment to strengthen semantic consistency between heterogeneous representations through mutual attention [52]. More recently, the integration of large language models with cross-view contrastive learning has demonstrated that high-level semantic reasoning provided by LLMs can effectively guide cross-modal representation learning and improve robustness under heterogeneous sensing conditions [53]. Although these methods achieve remarkable performance in feature alignment and multimodal representation learning, their primary objective is to maximize semantic consistency across different modalities. Li et al. proposed a framework for validating the behavioral consistency of large-language-model agents in simulated stock markets and analyzed whether their decision styles are consistent with those of real market participants [54]. Similarly, Biancotti et al. explored artificial-intelligence ethical alignment in financial decision-making by testing whether large language models, when acting as chief executive officers of financial institutions, behave in accordance with fiduciary duties and ethical standards [55]. These studies indicate that language–behavior consistency is not only valuable for state identification of real-world entities but is also becoming a new issue in artificial intelligence agent behavior evaluation and trustworthy intelligent systems research. The main mathematical notations used throughout this study are summarized in Table 1.

## 3. Materials and Methods

### 3.1. Data Collection

To construct a multisource dataset for the language–behavior consistency sensing task, data were collected from three levels, namely, textual sensing nodes, behavioral sensing nodes, and state-response nodes, as shown in Table 2. The data collection period ranged from January 2021 to December 2025, covering different stages such as stable system operation, intensified external disturbances, increased behavioral fluctuations, and potential risk exposure, so that good temporal continuity and scenario coverage could be ensured. The data collection work was mainly deployed at data acquisition nodes in information-intensive regions, including Beijing, Shanghai, Shenzhen, Hangzhou, and Guangzhou. Multisource heterogeneous data were continuously obtained through distributed data collection pipelines, behavioral data collection pipelines, and information collection pipelines. Specifically, the Beijing node was mainly responsible for collecting rule documents, public notifications, and management information flows; the Shanghai node was mainly responsible for collecting operational state records and high-frequency behavioral logs; the Shenzhen node was mainly responsible for collecting entity disclosure information and dynamic update information; the Hangzhou node was mainly responsible for collecting Internet information flows and media content; and the Guangzhou node was mainly responsible for collecting external response events and state feedback information. All nodes were coordinated through a unified time synchronization server, and standardized data interfaces were adopted for cross-node data transmission and aggregation. During collection, full synchronization was performed every 24 h, and incremental updates were performed every 1 h, thereby ensuring the temporal consistency and completeness of textual sensing signals, behavioral sensing signals, and state-response signals.

Textual sensing signals were mainly obtained from public information release nodes, organizational operation report nodes, policy and rule release nodes, announcement and notification nodes, and media information-flow nodes. These nodes continuously generated unstructured texts related to entity objectives, operational constraints, risk alerts, state descriptions, and future plans. During data acquisition, an automated textual sensing pipeline periodically collected data from web interfaces, application programming interfaces, and document repositories. Since the raw texts contained many tables, attachment descriptions, formatting noise, and repeated paragraphs, format parsing, deduplication, paragraph segmentation, and irrelevant-content filtering were performed after collection. Only core semantic segments related to objective declarations, constraint conditions, risk attitudes, resource allocation, and execution plans were retained, resulting in a textual sensing signal set covering multiple entities, time periods, and text-source types.

Behavioral sensing signals were mainly obtained from structured state-record nodes, operational-indicator monitoring nodes, behavioral event-log nodes, resource allocation record nodes, and external response monitoring nodes. The collected variables included continuous or discrete observations such as entity scale changes, resource flows, behavioral intensity, execution frequency, structural adjustment, state fluctuation, and external response. Compared with textual signals, behavioral signals have stronger temporal and numerical structural characteristics. Therefore, sampling was performed at daily, weekly, and monthly scales, and each behavioral observation was recorded using a unified timestamp mechanism. For high-frequency behavioral data, time-window aggregation was first conducted, and statistical features such as mean values, fluctuations, growth rates, extremes, and changing trends were extracted. For low-frequency structured data, completion and alignment were performed according to the observation period, so that behavioral variables from different sources could be incorporated into a unified time-series modeling framework. State-response signals were mainly obtained from anomaly record nodes, risk alert nodes, and external feedback nodes, and were used to mark whether state anomalies, enhanced fluctuations, or execution deviations occurred in subsequent windows. Finally, the release time of textual data was used as an anchor point, and behavioral sequences within fixed observation windows before and after the release time were synchronously matched with state-response results to generate language–behavior paired samples. This pipeline established explicit temporal associations among textual states, behavioral executions, and subsequent response states, providing a reliable foundation for consistency modeling and downstream risk analysis.

To improve the transparency and reproducibility of this study, a unified data construction pipeline was adopted to generate the final language–behavior paired samples. Specifically, the publication time of each textual sensing signal was used as the temporal anchor, and behavioral sequences within predefined observation windows were synchronously aligned with subsequent state-response records to construct supervised samples. All raw data underwent format parsing, duplicate removal, temporal synchronization, and cross-node consistency processing to ensure a unified temporal reference and consistent data structure across heterogeneous sources. Furthermore, all experiments were conducted using fixed random seeds, identical data partitioning strategies, and consistent training configurations. Since the final dataset was reconstructed by integrating publicly available textual information with continuously collected behavioral monitoring records, this study emphasizes a fully documented data construction pipeline and experimental configuration to facilitate reproducibility of the proposed framework. The proposed dataset is organized using a unified multisource sensing paradigm rather than being tailored to any individual application domain. It abstracts the general relationships among textual states, executed states, and state responses across heterogeneous sensing environments. Therefore, the reported experiments are intended to validate the general modeling capability of the proposed framework instead of evaluating domain-specific performance.

### 3.2. Data Augmentation

To improve the generalization capability of the proposed framework across different entities, observation windows, and operational environments, data augmentation was performed for textual sensing signals, behavioral time-series signals, structured state variables, and cross-modal relationships. Text augmentation preserves declared-state semantics; behavioral augmentation maintains execution-state dynamics; structured-data augmentation alleviates class imbalance; and cross-modal augmentation enhances language–behavior consistency learning. For textual sensing signals, back translation and paraphrase generation were adopted. Back translation generates alternative linguistic expressions while preserving the original state semantics:

(1) $$x^{bt}=T_{b\to a}\left(T_{a\to b}\left(x\right)\right),$$

where *x* denotes the original text, and $T_{a\to b}\left(\cdot \right)$ and $T_{b\to a}\left(\cdot \right)$ denote forward and backward translation, respectively. Semantic consistency is enforced by

(2) $$\mathrm{sim}\left(f_{text}\left(x\right),f_{text}\left(x^{bt}\right)\right)\geq \tau ,$$

where $f_{\mathrm{text}}\left(\cdot \right)$ denotes the text encoder, and τ is the semantic consistency threshold. Paraphrase generation directly produces semantically equivalent expressions,

(3) $$x^{pg}=G_{\theta }\left(x,z\right),$$

where $G_{\theta }\left(\cdot \right)$ is the paraphrase model, and *z* denotes random perturbations. The semantic difference before and after augmentation is measured as

(4) $$D_{state}\left(x,x^{pg}\right)={∥f_{text}\left(x\right)-f_{text}\left(x^{pg}\right).∥}_{2}.$$

Behavioral time-series augmentation includes noise perturbation and window slicing. Noise perturbation is defined as

(5) $${\tilde{b}}_{t}=b_{t}+\epsilon_{t},\;\epsilon_{t}∼N\left(0,\sigma^{2}I\right),$$

while the behavioral representation is constrained by

(6) $${∥f_{beh}\left(B\right)-f_{beh}\left(\tilde{B}\right)∥}_{2}\leq \delta ,$$

where δ denotes the allowable perturbation bound. Window slicing extracts local behavioral patterns,

(7) $$B_{s:s+l-1}=\left(b_{s},b_{s+1},\ldots ,b_{s+l-1}\right),\;1\leq s\leq T-l+1,$$

where *l* is the window length. For structured state variables, SMOTE and ADASYN were adopted to alleviate class imbalance. SMOTE generates synthetic samples by

(8) $$x_{new}=x_{i}+\lambda \left(x_{nn}-x_{i}\right),\;\lambda ∼U\left(0,1\right),$$

while ADASYN adaptively allocates synthetic samples according to

(9) $$g_{i}={\hat{r}}_{i}G.$$

For cross-modal augmentation, positive and negative language–behavior pairs were constructed with similarity

(10) $$s\left(x,B\right)=\mathrm{sim}\left(h_{x},h_{b}\right)=\frac{h_{x}^{⊤}h_{b}}{∥h_{x}∥\;∥h_{b}∥},$$

and optimized using the contrastive loss

(11) $$L_{con}=-\log\frac{\exp(\mathrm{sim}\left(h_{x},h_{b}^{+}\right)/\tau )}{\exp\left(\mathrm{sim}\left(h_{x},h_{b}^{+}\right)/\tau \right)+\sum_{k=1}^{K}\exp\left(\mathrm{sim}\left(h_{x},h_{b,k}^{-}\right)/\tau \right)}.$$

Finally, modality dropout is employed,

(12) $$h=\phi (m_{x}h_{x},m_{b}h_{b}),$$

where $m_{x}$ and $m_{b}$ are Bernoulli random variables indicating whether the textual and behavioral modalities are retained. This augmentation strategy improves the robustness of the proposed framework against modality perturbation and incomplete observations.

### 3.3. Proposed Method

#### 3.3.1. Overall

The proposed method is designed as a three-stage, multi-module language–behavior consistency sensing framework. The processed textual sensing signals and behavioral sensing signals are first fed into the textual state logic extraction module and the observed behavioral state modeling module, respectively. In the former module, unstructured texts such as public declaration texts, operation reports, risk alerts, and rule notifications are converted into multidimensional structured state logic vectors by using a large language model, so that core dimensions such as stability, extensibility, resource dependence, risk constraints, and transparency can be characterized. In the latter module, behavioral indicators, resource-flow records, event logs, and state-response sequences are modeled through time-series or tabular modeling structures, and behavioral state vectors reflecting execution intensity, state fluctuation, and potential risk exposure are generated. The two state vectors are then fed into the language–behavior consistency measurement module, where semantic matching, state conflict, and associations with subsequent abnormal events are measured in a unified representation space to produce an integrated consistency score. This score reflects the matching degree between textual states and actual executed states and can also be used for subsequent risk perception, anomaly warning, and state interpretation. In the overall workflow, the textual state vector provides the declaration-side reference for behavioral state modeling, while the behavioral state vector provides execution-side verification for consistency judgment. The consistency measurement module integrates the two through a multilayer interaction mechanism, thereby forming a closed-loop modeling process from textual perception and behavioral characterization to consistency quantification.

#### 3.3.2. Stated State Logic Extraction Module

The textual state logic extraction module aims to transform unstructured textual sensing signals into unified and interpretable state logic representations that serve as the textual reference for subsequent behavioral state modeling and language–behavior consistency measurement.

**Figure 1:**
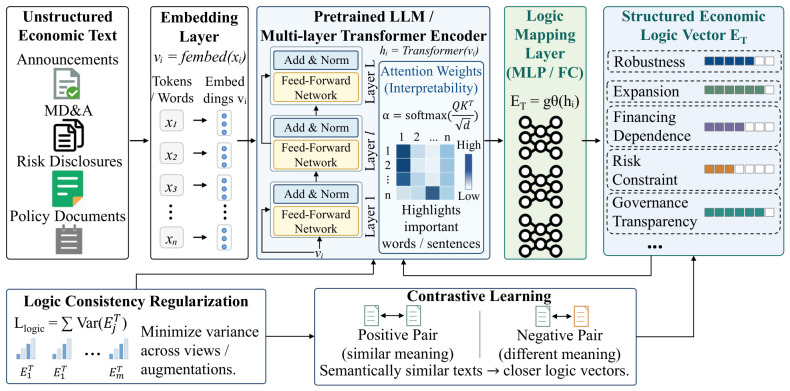
Architecture of the textual state logic extraction module, which transforms unstructured textual sensing signals into structured state logic representations through large language modeling, logic mapping, and contrastive regularization.

As illustrated in Figure 1, the module consists of a pretrained large language model, a Transformer encoder, and a logic mapping network. Given an input text

(13) $$X=x_{1},x_{2},\ldots ,x_{n},$$

where $x_{i}$ denotes the *i*-th text segment, the input is first encoded into contextual semantic embeddings

(14) $$v_{i}=f_{\mathrm{embed}}\left(x_{i}\right),$$

where $f_{\mathrm{embed}}\left(\cdot \right)$ denotes the pretrained text encoder. The embedding sequence is then processed by a Transformer encoder,

(15) H=Transformer(V),

where *V* is the embedding sequence, and *H* is the contextual representation. Self-attention weights are computed as

(16) $$A=\mathrm{softmax}\left(\frac{QK^{⊤}}{\sqrt{d}}\right),$$

and the contextual representation is obtained by

(17) H=AV,

where *Q*, *K*, and *V* denote the query, key, and value matrices, respectively. The contextual features are subsequently projected into the state logic space through a logic mapping network,

(18) $$E_{T}=g_{\theta }\left(H\right),$$

where $g_{\theta }\left(\cdot \right)$ denotes the logic mapping function, and $E_{T}$ is the textual state logic representation. To improve representation stability, a logic consistency regularization term is introduced,

(19) $$L_{\mathrm{logic}}=\sum_{j}\mathrm{Var}\left(E_{T}^{\left(j\right)}\right),$$

where $E_{T}^{\left(j\right)}$ denotes the representation of the *j*-th state logic dimension. The overall optimization objective is

(20) $$L_{T}=L_{\mathrm{rep}}+\alpha L_{\mathrm{logic}}+\beta L_{\mathrm{con}},$$

where α and β are weighting coefficients. Here, the three optimization objectives play complementary roles. The representation learning loss $L_{\mathrm{rep}}$ serves as the primary supervision objective and guides the textual state representation toward the downstream language–behavior consistency task. The logic consistency regularization $L_{\mathrm{logic}}$ minimizes the representation variance among semantically equivalent textual samples generated through semantic-preserving augmentations, thereby improving representation stability and robustness. However, this regularization alone does not explicitly enhance the discriminative capability of the learned representations. The discriminative property is mainly introduced by the contrastive learning objective $L_{\mathrm{con}}$, where positive pairs are constructed from semantically equivalent augmented texts and negative pairs are sampled from semantically inconsistent textual samples. By simultaneously minimizing the distance between positive pairs and maximizing the separation between negative pairs in the representation space, the contrastive objective enables the model to distinguish conflicting state representations while preserving semantic consistency among logically equivalent samples. Therefore, $L_{\mathrm{rep}}$, $L_{\mathrm{logic}}$, and $L_{\mathrm{con}}$, respectively, contribute task supervision, representation stability, and discriminative representation learning. Overall, the proposed module exploits the semantic reasoning capability of large language models to generate robust and interpretable textual state representations for subsequent consistency estimation.

#### 3.3.3. Observed Behavioral State Modeling Module

The observed behavioral state modeling module aims to extract execution-state representations from multidimensional behavioral sensing signals and project them into the same representation space as textual states.

**Figure 2:**
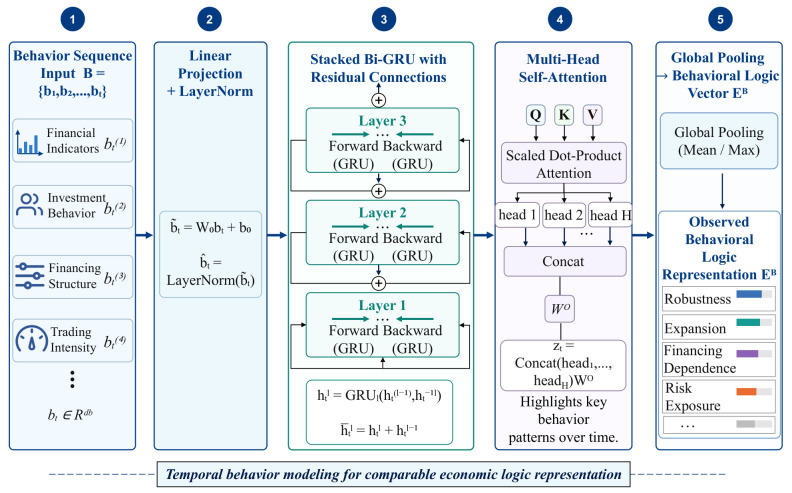
Architecture of the observed behavioral state modeling module, where multidimensional behavioral sensing sequences are encoded into behavioral logic representations using stacked Bi-GRU networks and multi-head self-attention mechanisms.

As illustrated in Figure 2, the module employs stacked bidirectional GRUs together with multi-head self-attention to capture temporal dependencies and informative behavioral patterns. Given the behavioral sequence

(21) $$B=\{b_{1},b_{2},\ldots ,b_{T}\},$$

where $b_{t}\in R^{d_{b}}$ denotes the behavioral feature vector at time step *t*, including resource flows, execution intensity, behavioral events, and state responses, the input is first projected into a latent feature space through linear transformation and normalization,

(22) $${\tilde{b}}_{t}=W_{0}b_{t}+b_{0},\;{\hat{b}}_{t}=\mathrm{LayerNorm}\left({\tilde{b}}_{t}\right).$$

The normalized features are subsequently encoded using stacked bidirectional GRUs,

(23) $$s_{t}^{\left(l\right)}={\mathrm{GRU}}_{l}\left(s_{t-1}^{\left(l\right)},s_{t}^{\left(l-1\right)}\right),$$

(24) $${\overset{\leftarrow }{s}}_{t}^{\left(l\right)}={\mathrm{GRU}}_{b}^{\left(l\right)}\left({\overset{\leftarrow }{s}}_{t+1}^{\left(l\right)},s_{t}^{\left(l-1\right)}\right),$$

where the bidirectional hidden representation is obtained by

(25) $$s_{t}^{\left(l\right)}=\left[{\vec{s}}_{t}^{\left(l\right)};{\overset{\leftarrow }{s}}_{t}^{\left(l\right)}\right].$$

Multi-head self-attention $N_{h}$ is then applied to emphasize informative behavioral patterns. For the *m*-th head,

(26) $${\mathrm{head}}^{\left(m\right)}=\mathrm{Attention}\left(Q^{\left(m\right)},K^{\left(m\right)},V^{\left(m\right)}\right),$$

and the final behavioral representation is computed as

(27) $$z_{t}=\mathrm{Concat}\left({\mathrm{head}}^{\left(1\right)},\ldots ,{\mathrm{head}}^{\left(N_{h}\right)}\right)W^{O}.$$

where $W_{O}$ is the output projection matrix. Finally, global average pooling aggregates temporal information into the behavioral state representation,

(28) $$E_{B}=\frac{1}{T}\sum_{t=1}^{T}z_{t}.$$

where $E_{B}$ denotes the behavioral state vector over the entire observation period. By combining stacked Bi-GRU networks with multi-head self-attention, the proposed module effectively captures both long-term temporal dependencies and salient behavioral patterns, producing expressive behavioral state representations.

#### 3.3.4. Language–Behavior Consistency Measurement Module

**Figure 3:**
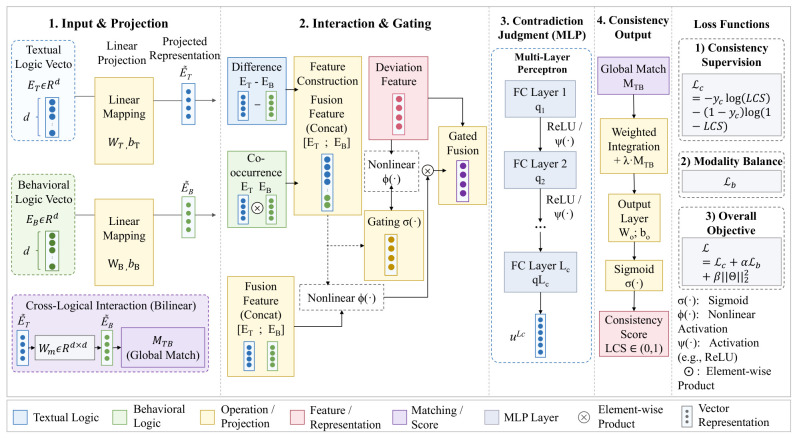
Architecture of the language–behavior consistency measurement module, which evaluates the consistency between declared logic and observed behavior through cross-logical interaction, gated discrepancy learning, and consistency-aware prediction.

The language–behavior consistency measurement module quantifies the consistency between declared and executed states in a unified state representation space. As illustrated in Figure 3, the textual state representation and behavioral state representation are denoted by $E_{T}$ and $E_{B}$, respectively.

The bilinear interaction term is employed to compute a global compatibility score between the textual state representation and the behavioral state representation, summarizing the overall semantic agreement between the two representations and serves as a global interaction signal for subsequent consistency estimation. Detailed discrepancy learning and dimension-wise information fusion are performed by the subsequent difference feature construction, gating mechanism, and multilayer perceptron, which jointly capture local semantic inconsistencies and complementary information across the two representation spaces.

(29) $$M_{TB}=E_{T}^{⊤}W_{m}E_{B}+b_{m},$$

where $W_{m}$ is the learnable interaction matrix, and $b_{m}$ is the bias term. Difference features and synergistic features are then jointly constructed and fused through a gating mechanism,

(30) $$D=\phi \left(W_{d}\left[E_{T}-E_{B};\;E_{T}⊙E_{B}\right]+b_{d}\right),$$

(31) $$G=\sigma \left(W_{g}D+b_{g}\right),$$

(32) $$F=G⊙D+\left(1-G\right)⊙\phi (W_{f}\left[E_{T};E_{B}\right]+b_{f}),$$

where *D* denotes the discrepancy representation, *G* denotes the gating weights, and *F* is the fused language–behavior representation. The fused representation is subsequently processed by a multilayer perceptron,

(33) $$u^{\left(l\right)}=\psi \left(W^{\left(l\right)}u^{\left(l-1\right)}+b^{\left(l\right)}\right),\;u^{\left(0\right)}=F,$$

and the final language–behavior consistency score is computed as

(34) $$LCS=\sigma \left(W_{o}u^{\left(L_{c}\right)}+b_{o}+\lambda M_{TB}\right),$$

The model is optimized using the consistency supervision loss,

(35) $$L_{c}=-y_{c}\log\left(LCS\right)-\left(1-y_{c}\right)\log\left(1-LCS\right)$$

where $y_{c}\in \left\{0,1\right\}$ denotes the language–behavior consistency label, wherein $y_{c}=1$ indicates a consistent language–behavior pair and $y_{c}=0$ indicates an inconsistent pair. To improve the mathematical stability of the optimization objective, the textual and behavioral state representations are first normalized using $\ell_{2}$ normalization before modality alignment is performed:

(36) $${\hat{E}}_{T}=\frac{E_{T}}{∥E_{T}{∥}_{2}},\;{\hat{E}}_{B}=\frac{E_{B}}{∥E_{B}{∥}_{2}}.$$

The modality balance objective is then defined as

(37) $$L_{b}={∥W_{T}{\hat{E}}_{T}-W_{B}{\hat{E}}_{B}∥}_{2}^{2}+\eta \left(1-\frac{{\hat{E}}_{T}^{⊤}{\hat{E}}_{B}}{∥{\hat{E}}_{T}{∥}_{2}{∥{\hat{E}}_{B}∥}_{2}}\right),$$

where the cosine-distance regularization is naturally bounded and encourages compatible representations between textual and behavioral modalities. Since all embeddings are normalized during training, the optimization objective remains numerically stable while preventing representation collapse. This regularization improves modality alignment, whereas discriminative representation learning is primarily achieved by the contrastive learning objective introduced in the textual state representation module. Leading to the overall objective

(38) $$L=L_{c}+\alpha L_{b}+\beta {∥\Theta ∥}_{2}^{2},$$

where Θ denotes the learnable parameters. The proposed framework is trained by jointly minimizing the overall objective. Specifically, the optimal model parameters are obtained by

(39) $$\Theta^{*}=\arg\min_{\Theta }\left(L_{c}+\alpha L_{b}+\beta {∥\Theta ∥}_{2}^{2}\right),$$

where all three objective terms are simultaneously optimized through backpropagation using the AdamW optimizer. The consistency supervision loss $L_{c}$ provides the primary learning signal for language–behavior consistency prediction, the modality balance loss $L_{b}$ encourages compatible representations between textual and behavioral state spaces, and the $\ell_{2}$ regularization term improves model generalization by suppressing parameter overfitting. Therefore, the gradients used for parameter updates are computed from the complete objective function rather than from the consistency score alone, ensuring that representation consistency, modality alignment, and model robustness are jointly optimized throughout the training process. The proposed module is optimized by jointly minimizing the consistency supervision loss, modality balance loss, and parameter regularization objective, enabling global interaction modeling, modality alignment, and robust representation learning within a unified optimization framework.

## 4. Results and Discussion

### 4.1. Experimental Configuration

#### 4.1.1. Hardware and Software Platform

In terms of hardware environment, the experiments were conducted on a server platform with strong parallel computing capability. The hardware configuration included an Intel Xeon multi-core processor (Intel, Santa Clara, CA, USA) with a main frequency of no less than 2.40 GHz and 128 GB of system memory, which was used to support large-scale textual sensing signal reading, behavioral state variable calculation, and caching during model training. An NVIDIA RTX 3090 GPU (Nvidia, Santa Clara, CA, USA) or an equivalent graphics processing unit with 24 GB of video memory was used to accelerate deep learning models and text representation computation related to large language models. The experimental data were stored on a high-speed solid-state drive with a capacity of no less than 2 TB, ensuring stable reading of textual sensing signals, behavioral sensing signals, and intermediate feature files during training and evaluation. In terms of software environment, the experimental platform was built on the Ubuntu 20.04 operating system, and Python 3.9 was used as the main programming language. PyTorch 1.13 was adopted as the deep learning framework, and the Transformers library was used for pretrained language model invocation, text encoding, and semantic representation extraction. Data processing was mainly performed using Pandas 1.5.3, NumPy 1.23.5, and scikit-learn 1.2.2 for sample cleaning, variable standardization, dataset partitioning, cross-validation, and evaluation metric calculation. CUDA 11.7 and cuDNN 8.5.0 were used to accelerate GPU computation during model training. Fixed random seeds were set for all experiments to ensure good reproducibility of the results. In terms of hyperparameter settings, the original samples were divided into training, validation, and test sets at ratios of 70%, 15%, and 15%, respectively. The training set was used for model parameter learning, the validation set was used for hyperparameter tuning and early stopping, and the test set was used only for final performance evaluation. To further reduce the influence of randomness in sample partitioning on experimental results, five-fold cross-validation was adopted. Specifically, all samples were divided into five non-overlapping subsets; one fold was selected as the validation set each time, and the remaining four folds were used as the training set. The averaged results of the five experiments were taken as the final results. The learning rate was set to $2\times 10^{-5}$, the batch size was set to 16, the maximum text length was set to 512, and the number of training epochs was set to 10. AdamW was used as the optimizer, with a weight decay coefficient of 0.01 and a Dropout rate of 0.1. In the calculation of the consistency score, the weights $w_{k}$ of each state-logic dimension were set equally by default, and the influence of different weight settings on the results was further examined in robustness tests.

To ensure fair, reliable, and reproducible evaluation, the entire dataset construction and partitioning procedure was carefully designed to eliminate potential information leakage. Duplicate textual records, repeated behavioral sequences, and identical language–behavior pairs were removed before data partitioning. All paired samples were generated through temporal alignment and manually verified to ensure annotation quality. The dataset was partitioned at the entity level, ensuring that samples originating from the same entity never appeared in different subsets. The initial training, validation, and test sets were divided in a 70%/15%/15% ratio. Five-fold cross-validation was performed exclusively within the training set for model selection and hyperparameter tuning, while the independent test set remained untouched until the final evaluation. For the unseen-entity evaluation, all entities in the test set were excluded from the training process. For the unseen-scenario evaluation, complete operational scenarios were held out according to temporal and environmental conditions to assess generalization under distribution shifts. Unless otherwise specified, all reported results are presented as the mean ± standard deviation over repeated runs, and paired *t*-tests (p<0.05) were conducted to verify the statistical significance of performance improvements.

#### 4.1.2. Baseline Models and Evaluation Metrics

To provide representative yet focused comparisons, this study selected two pretrained language models (PLMs) as text-based baselines rather than exhaustively evaluating all available PLMs. Specifically, FinBERT was chosen as a representative domain-specific PLM, while RoBERTa was selected as a representative general-purpose PLM. Together, these models cover the two major application paradigms of encoder-based pretrained language models and provide a meaningful benchmark for conventional text representation approaches. Other models, such as BERT, DeBERTa, ALBERT, and ELECTRA, also belong to the encoder-based PLM family and share similar modeling principles. Therefore, the primary objective of this work is not to compare different PLMs, but to demonstrate the advantages of the proposed LLM-based language–behavior consistency framework over conventional PLM-based text understanding methods. FinBERT [56], RoBERTa + MLP [57], XGBoost [58], LSTM [59], Rule-Based Consistency [60], Isolation Forest [61], Multimodal Transformer [62], and the Contrastive Learning Multimodal Model [63] were selected as baseline methods. FinBERT and RoBERTa+MLP were adopted as representative PLM baselines rather than large language models. Specifically, FinBERT is a domain-specific PLM optimized for financial text understanding, whereas RoBERTa is a general-purpose PLM pretrained on large-scale text corpora. These models were included to evaluate the capability of conventional PLM-based text representation learning and to provide a comparison with the LLM-based textual state logic extraction module employed in the proposed framework. XGBoost was used to process structured behavioral state variables and evaluate the applicability of traditional ensemble learning methods to state identification tasks. LSTM was used to characterize the temporal dependencies of behavioral sensing signals. Rule-Based Consistency determined whether textual declarations matched behavioral states according to manually designed rules. Isolation Forest was used to identify anomalous samples in behavioral data and compare the performance of conventional unsupervised anomaly detection methods. Multimodal Transformer jointly modeled the association between textual state representations and behavioral state representations. Contrastive Learning Multimodal Model learned the matching relationship between different modalities by constructing consistent and inconsistent language–behavior sample pairs. These baseline methods covered different technical routes, including text-only modeling, behavior-only modeling, rule-based judgment, anomaly detection, and multimodal fusion, thereby enabling a comprehensive evaluation of the proposed method.

The model was comprehensively evaluated from five perspectives: consistency sensing capability, abnormal state identification capability, classification discrimination performance, explanation reliability, and subsequent state association. Specifically, the Logic Consistency Score (LCS), AUC, Precision, Recall, F1-score, Explanation Consistency Score (ECS), and a state-response association model were adopted as the main evaluation criteria. The corresponding formulas are given as follows:

(40) $$LCS_{i,t}=1-\frac{\sum_{k=1}^{K}w_{k}\left|S_{i,t}^{k}-B_{i,t}^{k}\right|}{\sum_{k=1}^{K}w_{k}},$$

(41) $$AUC=\int_{0}^{1}TPR\left(FPR^{-1}\left(u\right)\right)du,$$

(42) $$Precision=\frac{TP}{TP+FP},$$

(43) $$Recall=\frac{TP}{TP+FN},$$

(44) $$F1=\frac{2\times Precision\times Recall}{Precision+Recall},$$

(45) $$ECS=\frac{1}{N}\sum_{i=1}^{N}I\left({\hat{D}}_{i}=D_{i}^{case}\right),$$

(46) $$Risk_{i,t+h}=\alpha +\beta \left(1-LCS_{i,t}\right)+\gamma X_{i,t}+\mu_{i}+\lambda_{t}+\varepsilon_{i,t}.$$

To improve the transparency and reproducibility of the evaluation protocol, all consistency-related metrics are normalized to the interval [0,1], where higher values indicate better performance. Specifically, the Language–Behavior Consistency Score (LCS) measures the normalized agreement between textual state representations and behavioral state representations. The Explanation Consistency Score (ECS) is calculated as the proportion of samples whose explanation direction predicted by the proposed framework agrees with expert annotations. For ECS evaluation, three researchers with experience in intelligent sensing independently annotated randomly selected language–behavior sample pairs as either “consistent” or “inconsistent” according to whether the observed behavioral evolution matched the corresponding textual declaration. Disagreements were resolved through discussion until consensus was reached. The resulting consensus annotations were used exclusively as the reference labels for ECS computation. Furthermore, the risk model in Equation (42) was introduced as an auxiliary statistical analysis rather than an additional prediction model. It was employed to examine whether lower language–behavior consistency is associated with increased subsequent anomaly risk. All behavioral variables used in the regression analysis were standardized before estimation, while individual and temporal fixed effects were included to improve the robustness and interpretability of the statistical analysis. Here, $LCS_{i,t}$ denotes the language–behavior consistency score of entity *i* at time *t*. $S_{i,t}^{k}$ and $B_{i,t}^{k}$ denote the *k*-th textual declared-state dimension and behavioral executed-state dimension, respectively. $w_{k}$ is the dimension weight, and *K* is the total number of state dimensions. AUC is calculated from TPR and FPR, while Precision, Recall, and F1-score are determined by TP, FP, and FN. ECS denotes the explanation consistency score, ${\hat{D}}_{i}$ denotes the deviation direction output by the model, $D_{i}^{case}$ denotes the case-based or expert-annotated direction, and *N* denotes the number of samples. $Risk_{i,t+h}$ denotes the abnormal state of entity *i* in the future period *h*, $X_{i,t}$ denotes control variables, $\mu_{i}$ and $\lambda_{t}$ denote individual fixed effects and time fixed effects, respectively, and $\varepsilon_{i,t}$ denotes the error term.

### 4.2. Performance Comparison with Baseline Methods

This experiment was designed to evaluate the overall effectiveness of the proposed language–behavior consistency sensing framework in multisource sensing environments and to compare its performance with representative text-based models, behavior-based models, rule-driven methods, anomaly detection approaches, and multimodal fusion models. The effectiveness of different methods was assessed from three perspectives, namely, consistency measurement capability (LCS), anomaly identification capability (AUC, F1, and Precision), and interpretability consistency (ECS).

**Figure 4:**
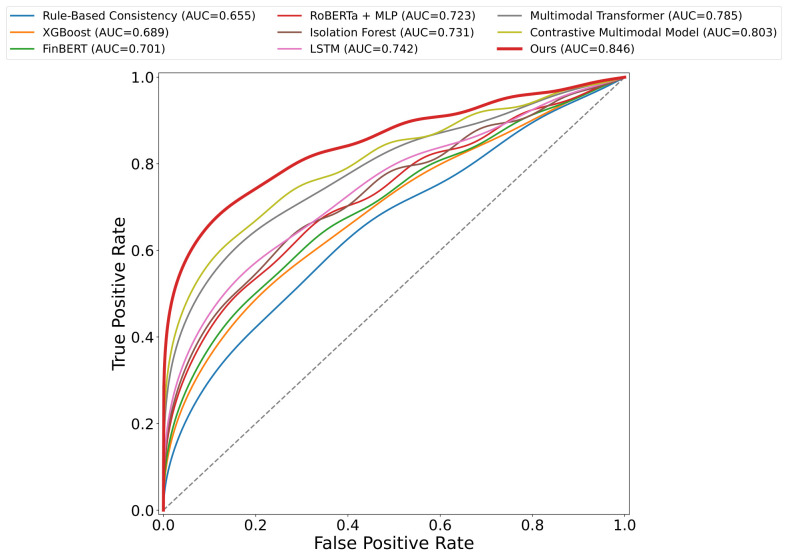
ROC curves of different baseline methods and the proposed framework, demonstrating that the proposed model achieves the highest discrimination capability with an AUC of 0.846.

As shown in Table 3 and Figure 4, the overall performance of single-modal text models and behavior models was relatively limited. As representative PLM-based text models, FinBERT and RoBERTa+MLP were capable of extracting declaration-state information from textual sensing signals and therefore achieved better LCS and ECS than conventional structured-data models. However, because these PLM-based approaches relied solely on textual semantic representations without modeling behavioral execution information, their capability for subsequent anomaly identification remained limited. XGBoost relied solely on structured behavioral variables and was able to capture nonlinear relationships among features, but it lacked the capability to model the interaction between textual states and executed states, resulting in relatively weak overall performance. LSTM utilized recurrent temporal structures to model dynamic dependencies within behavioral sequences and therefore outperformed text-based models in terms of AUC and F1, indicating that behavioral execution states contain substantial information relevant to anomaly warning. Although Rule-Based Consistency achieved an ECS value of 0.701, suggesting relatively strong agreement with expert knowledge, its limited representational capacity prevented adaptation to diverse state logic patterns in complex scenarios, leading to the weakest overall detection performance. Isolation Forest identified statistically abnormal samples and achieved an AUC of 0.731; however, its focus on statistical deviations rather than declaration–execution relationships resulted in only moderate performance on consistency-related metrics.

As multimodal modeling capabilities increased, a clear improvement trend was observed across all evaluation metrics. The Multimodal Transformer simultaneously modeled textual and behavioral sensing signals and learned cross-modal associations through self-attention mechanisms, thereby significantly outperforming all unimodal approaches. The Contrastive Multimodal Model further introduced contrastive constraints to optimize the representation relationships between consistent and inconsistent samples, resulting in additional improvements in LCS, AUC, and ECS, which demonstrates the importance of cross-modal semantic alignment for consistency sensing. Nevertheless, these methods mainly emphasize modality fusion or feature alignment and do not explicitly model the logical deviation mechanism between textual states and executed states. In contrast, the proposed framework achieved the best performance across all metrics, reaching 0.742, 0.846, 0.811, 0.802, and 0.821 in terms of LCS, AUC, F1, Precision, and ECS, respectively. From a theoretical perspective, the declared-state logic extraction module utilizes large language models to construct high-level semantic logic representations; the behavioral-state modeling module captures long-term behavioral evolution patterns; and the consistency measurement module explicitly characterizes the matching and conflicting relationships between the two state spaces through cross-state interaction, difference gating, and conflict discrimination mechanisms. Compared with simple feature concatenation or attention-based fusion strategies, the proposed architecture not only preserves the representational capabilities of textual and behavioral information but also strengthens both cooperative and conflicting characteristics between them. From a mathematical perspective, the multilayer interaction structure enhances nonlinear representation capacity in high-dimensional latent spaces, the gating mechanism increases sensitivity to critical discrepancy dimensions, and the consistency supervision objective aligns representation learning with the final task objective. Consequently, superior detection performance, stronger interpretability, and more stable cross-scenario generalization capability are achieved.

### 4.3. Generalization Performance Under Unseen Settings

This experiment was conducted to evaluate the generalization capability of different models under unseen scenarios, unseen entities, and distribution-shift conditions. The primary objective was to investigate whether transferable language–behavior association patterns could be learned, rather than merely memorizing local patterns within the training data.

**Figure 5:**
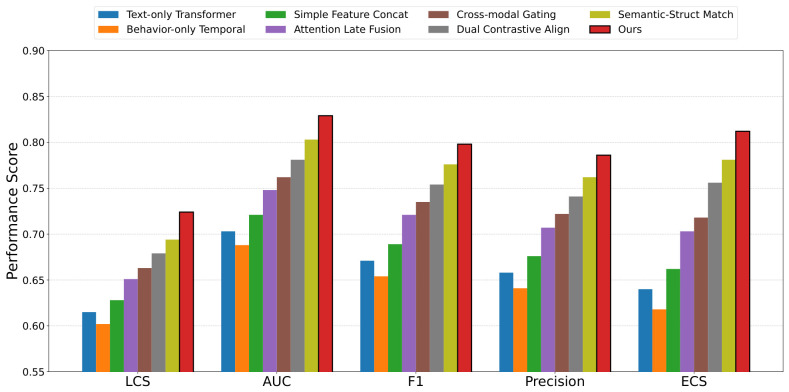
Generalization performance of different models under unseen settings across five evaluation metrics, where the proposed framework consistently achieves the best overall performance.

As shown in Table 4 and Figure 5, the overall performance of unimodal models remained relatively limited. The Text-only Transformer Encoder relied exclusively on textual sensing signals for state modeling and was capable of capturing semantic information to a certain extent, resulting in an LCS of 0.615 and an AUC of 0.703. However, its performance under cross-scenario conditions was sensitive to variations in textual expression due to the lack of behavioral evidence. The Behavior-only Temporal Encoder modeled only behavioral sequences and learned temporal dynamic characteristics but lacked declaration-state information, resulting in slightly lower performance than the text-based model across all metrics. Simple Feature Concatenation achieved moderate improvements by directly combining textual and behavioral features; however, its fusion strategy remained at the feature level and was unable to capture complex cross-modal dependencies. The Attention-based Late Fusion Model dynamically assigned modality weights through attention mechanisms and improved the AUC to 0.748, indicating that attention-based feature selection can enhance the extraction of informative signals. The Cross-modal Gating Network further regulated information flow across modalities using gating structures, effectively reducing noise interference and improving LCS, F1, and ECS. Overall, a stable improvement trend was observed as increasingly sophisticated cross-modal interaction mechanisms were incorporated, highlighting the importance of declaration-state and execution-state relationships for state identification in unseen environments.

Further analysis of the high-performing models reveals that the Dual-stream Contrastive Alignment Model and the Semantic-Structural Matching Network achieved AUC values of 0.781 and 0.803, respectively, significantly outperforming conventional fusion methods. The former leveraged contrastive learning to optimize distances between modalities within a shared representation space, thereby clustering consistent samples and separating inconsistent samples, which resulted in more stable cross-domain representations. The latter further incorporated matching relationships between semantic and structural information, leading to stronger state-logic representations and improved LCS and ECS values of 0.694 and 0.781, respectively. Nevertheless, these methods primarily focus on representation alignment or feature matching and lack explicit modeling of the deviation mechanism between declared and executed states. In contrast, the proposed framework achieved the best performance across all metrics, reaching 0.724, 0.829, 0.798, 0.786, and 0.812 in terms of LCS, AUC, F1, Precision, and ECS, respectively. The declared-state representations generated by large language models provide strong semantic abstraction capabilities, the behavioral-state modeling module captures long-term temporal dependencies, and the consistency measurement module explicitly characterizes cooperative and conflicting relationships between the two state spaces through cross-state interaction, difference gating, and conflict discrimination mechanisms. From a mathematical perspective, unimodal models learn only local feature distributions, whereas simple fusion models mainly establish shallow or linear associations. The proposed framework employs multilayer nonlinear interactions to model higher-order relationships, enabling state consistency and state conflict to be effectively separated within the latent space. Furthermore, the gating mechanism dynamically emphasizes critical discrepancy dimensions while suppressing irrelevant features, thereby maintaining strong discriminative ability and interpretability under unseen entities and scenarios and demonstrating superior generalization and environmental adaptability.

### 4.4. Ablation Study

This experiment was designed to verify the contribution of each core component within the proposed framework and to further reveal the functional roles of different modules in the language–behavior consistency sensing process.

**Figure 6:**
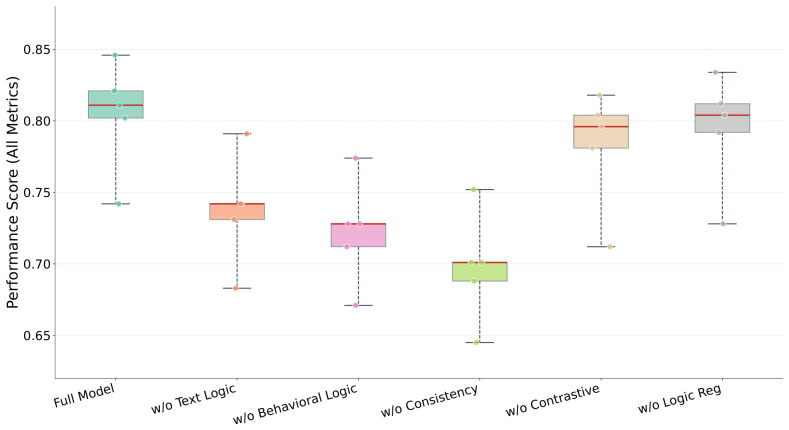
Boxplot visualization of ablation study results, illustrating the contribution of each module to the overall performance and the superiority of the complete framework.

As shown in Table 5 and Figure 6, the full model achieved the best performance across all evaluation metrics, with LCS, AUC, F1, Precision, and ECS values of 0.742, 0.846, 0.811, 0.802, and 0.821, respectively, indicating a clear synergistic effect among all components. When the text-logic module was removed, all metrics decreased substantially, with AUC dropping to 0.791 and ECS decreasing to 0.742, demonstrating the importance of declaration-state information for consistency modeling. The text-logic module extracts high-level semantic state information from unstructured textual signals and provides a reference basis for subsequent consistency judgment. Without this module, the model relies exclusively on behavioral features, resulting in a noticeable reduction in interpretability. Removing the behavioral-logic module caused a further decline in performance, with AUC decreasing to 0.774, indicating that actual execution states constitute another essential source of information for consistency assessment. Textual signals describe declared intentions, whereas behavioral signals reflect actual execution processes, making both modalities indispensable. Among all variants, the removal of the consistency module resulted in the most significant performance degradation, with LCS decreasing to 0.645, AUC to 0.752, and F1 to 0.701. This observation suggests that simply obtaining textual and behavioral features is insufficient for consistency sensing; rather, the critical factor lies in explicitly modeling the matching and conflicting relationships between them. The consistency module therefore serves as the core component responsible for cross-modal relationship learning and acts as the primary bridge connecting textual statesand executed states.

Further analysis of the contrastive learning and logic regularization components reveals that, although these modules are not the principal structural components, they significantly improve training stability and generalization capability. After removing contrastive learning, AUC decreased from 0.846 to 0.818, and ECS declined from 0.821 to 0.804, indicating that contrastive learning effectively clusters consistent samples while separating inconsistent samples within the representation space, thereby improving class discrimination. From a mathematical perspective, contrastive learning optimizes the distribution structure of latent representations, reduces inter-class overlap, and promotes more stable cross-modal correspondences. Removing logic regularization produced a relatively smaller performance decrease; however, all metrics remained lower than those of the full model, suggesting that regularization helps reduce the influence of noisy expressions and local outliers on state-logic representations while improving representation consistency across samples. Overall, the text-logic module and behavioral-logic module construct the declaration-state and execution-state spaces, respectively, while the consistency module learns the mapping relationship between them. Contrastive learning and logic regularization further optimize representation distributions and training stability. Because the full model simultaneously incorporates semantic abstraction, temporal modeling, cross-modal interaction, and representation regularization, it can more accurately distinguish state consistency from state conflict in high-dimensional feature spaces, resulting in superior anomaly detection performance and interpretability consistency. These findings further demonstrate that language–behavior consistency sensing is achieved through collaborative modeling and joint optimization across multiple components rather than relying on a single information source.

### 4.5. Discussion

The experimental results demonstrate that language–behavior consistency sensing not only improves anomaly-state identification performance but also provides a novel analytical perspective for risk perception and state interpretation in complex system operations. In practical industrial operation and maintenance scenarios, management units regularly release operation plans, maintenance strategies, safety specifications, and resource scheduling instructions, while operational systems continuously generate device logs, workload records, resource utilization statistics, and alarm information. Conventional monitoring approaches typically focus on behavioral indicators such as temperature, current, workload, and fault alarms while overlooking whether these behaviors remain consistent with declared operational strategies. For example, a data center may continuously emphasize energy-efficient operation and load-balancing strategies in official reports, whereas monitoring data indicate that certain servers operate under persistently high workloads and highly concentrated resource allocation. Although no obvious failures may have occurred, a deviation between textual states and executed states has already emerged. The proposed framework simultaneously analyzes textual declarations and behavioral responses, enabling potential risks to be identified before performance degradation or system failures become apparent and thereby providing earlier decision support for operators.

Similar situations frequently arise in intelligent manufacturing and industrial production environments, where production schedules, equipment operating specifications, and process-control requirements constitute textual sensing signals, while vibration, temperature, energy consumption, operating frequency, and production rhythm data collected by sensors constitute behavioral sensing signals. When operational documents require stable production rhythms and controlled energy consumption, yet actual operations exhibit frequent start–stop cycles, abnormal energy growth, or intensified workload fluctuations, such deviations may indicate equipment aging, process instability, or inadequate management execution. Comparable patterns can also be observed in smart campuses, cloud-platform operation and maintenance, network infrastructure management, and large-scale equipment cluster monitoring. Unlike conventional anomaly detection methods that focus solely on whether numerical values exceed predefined thresholds, the proposed framework emphasizes whether declared intentions are genuinely reflected in execution behavior. Consequently, many state patterns that have not yet evolved into extreme anomalies but already exhibit execution deviations can be effectively identified. The experimental results indicate that the proposed framework consistently outperforms baseline approaches in terms of consistency measurement, anomaly detection, and interpretability consistency. By constructing a unified language–behavior representation space, abnormal behaviors can not only be detected but also explained through their relationships with declared operational objectives, thereby improving the accuracy, interpretability, and practical value of complex system state perception.

### 4.6. Limitations and Future Work

Although the proposed language–behavior consistency sensing framework demonstrates promising effectiveness across multiple experiments, several limitations remain. First, consistency relationships are established primarily using publicly available textual sensing signals and observable behavioral sensing signals. In real-world complex systems, however, many latent decision factors, internal control information, and unobservable state variables cannot be directly accessed, which may lead to incomplete representations of textual states and executed states. Second, temporal lag effects often exist between textual declarations and behavioral responses, and the response cycles vary considerably across different entities and application scenarios. The current framework relies on relatively unified observation windows and therefore does not fully capture long-term lag dependencies or dynamic evolutionary processes. In addition, the present framework mainly focuses on consistency measurement between textual and behavioral modalities. Other information sources, such as images, videos, speech signals, and real-time Internet-of-Things sensing data, have not yet been integrated into a unified sensing architecture, which may limit global state-awareness capability in highly complex environments.

Future research may extend the framework in several directions. Dynamic temporal alignment mechanisms, causal inference techniques, and continuous state-evolution modeling approaches may be incorporated to better characterize long-term dependencies and transmission mechanisms between textual states and executed states, thereby improving the interpretability of risk formation processes. Furthermore, visual sensing information, network topology data, spatial-location information, and Internet-of-Things sensor streams may be integrated to establish a more comprehensive multimodal consistency sensing framework capable of capturing system states from a broader perspective. With the rapid advancement of large language models and multimodal foundation models, knowledge-enhanced reasoning and multi-agent collaborative understanding mechanisms may also be explored to improve the accuracy and generalization capability of state-logic extraction in complex environments. Such developments are expected to provide stronger intelligent sensing support for practical applications including industrial operation and maintenance, intelligent manufacturing, digital infrastructure management, and smart-city monitoring.

### 4.7. Discussion on Computational Complexity and Practical Deployment

The computational cost of the proposed framework is mainly introduced by the large language model during the textual state logic extraction stage, whereas the observed behavioral state modeling and language–behavior consistency measurement modules employ relatively lightweight neural architectures. In practical deployment, textual declarations are updated much less frequently than behavioral sensing signals. Therefore, textual state representations can be generated offline and cached, allowing online inference to perform only behavioral feature encoding and consistency prediction, thereby substantially reducing computational overhead. Furthermore, the proposed framework adopts a modular architecture in which textual signals, behavioral sequences, and state-response information are integrated through a unified state representation space. This design enables straightforward extension to additional sensing modalities and larger-scale datasets by incorporating corresponding feature encoding modules without modifying the overall framework. Consequently, the proposed method demonstrates favorable scalability and practical deployment potential for real-time intelligent monitoring and anomaly awareness applications.

## 5. Conclusions

This paper proposed a multisource language–behavior consistency sensing framework that integrates textual sensing signals, behavioral sensing signals, and state-response information to enable explainable perception of consistency between declared and executed states. The proposed framework consists of three core components: textual state logic extraction, observed behavioral state modeling, and language–behavior consistency measurement. By mapping heterogeneous sensing information into a unified state representation space, the proposed method effectively quantifies declaration–execution deviations and identifies potential anomalies. Experimental results demonstrate that the proposed framework consistently outperforms representative baseline methods in language–behavior consistency evaluation, anomaly awareness, and interpretability, confirming its effectiveness and robustness. Owing to its modular design, the framework is readily extensible to other multisource sensing scenarios. Future work will further evaluate the proposed framework on additional public datasets and diverse real-world application scenarios to validate its cross-domain generalization capability and investigate its potential applications in industrial operation and maintenance, intelligent manufacturing, digital infrastructure management, and other intelligent monitoring tasks.

## Figures and Tables

**Table 1:** Summary of main mathematical notations.

Notation	Meaning	Description
$X=\{x_{1},\ldots ,x_{n}\}$	Input textual sequence	Input textual sensing signal consisting of *n* text segments.
$B=\{b_{1},\ldots ,b_{T}\}$	Behavioral sequence	Behavioral sensing sequence over the observation window.
$E_{T}$	Textual state representation	Structured state representation extracted from textual sensing signals.
$E_{B}$	Behavioral state representation	Behavioral state representation learned from temporal behavioral signals.
${\hat{E}}_{T},{\hat{E}}_{B}$	Normalized representations	$\ell_{2}$-normalized textual and behavioral representations for modality alignment.
*H*	Contextual representation	Contextual semantic representation produced by the Transformer encoder.
$s_{t}^{\left(l\right)}$	Bi-GRU hidden state	Hidden representation at time step *t* in the *l*-th Bi-GRU layer.
$N_{h}$	Number of attention heads	Number of heads in the multi-head self-attention module.
$M_{TB}$	Global compatibility score	Global semantic compatibility obtained by bilinear interaction between $E_{T}$ and $E_{B}$.
LCS	Language–Behavior Consistency Score	Predicted consistency score between textual and behavioral states. Larger values indicate stronger consistency.
$y_{c}$	Consistency label	Binary supervision label, where $y_{c}=1$ denotes a consistent language–behavior pair and $y_{c}=0$ otherwise.
$L_{\mathrm{rep}}$	Representation learning loss	Primary supervision objective for textual representation learning.
$L_{\mathrm{logic}}$	Logic consistency regularization	Variance regularization preserving semantic consistency among augmented textual samples.
$L_{\mathrm{con}}$	Contrastive learning loss	Contrastive objective encouraging discriminative textual state representations.
$L_{c}$	Consistency supervision loss	Binary cross-entropy loss for language–behavior consistency prediction.
$L_{b}$	Modality balance loss	Bounded modality alignment objective computed in the normalized representation space.
*L*	Overall optimization objective	Joint optimization objective combining consistency supervision, modality alignment, and parameter regularization.
Θ	Trainable parameters	All learnable parameters of the proposed framework.

**Table 2:** Statistics of the multisource sensing dataset.

Data Type	Sensor Model	Sample Size
Public declaration texts	Public disclosure documents	520,000
Operation report texts	Corporate operation reports	360,000
Rule and notification texts	Public policy and notification documents	210,000
Media information-flow texts	Public news and media reports	340,000
Structured state records	Structured operational records	1,280,000
Behavioral event logs	Behavioral event records	960,000
Resource allocation records	Resource allocation records	740,000
External response records	External response records	620,000
Language–behavior paired samples	Integrated paired dataset	920,000

**Table 3:** Performance comparison with baseline methods. Improvements of the proposed method over the strongest baseline are statistically significant according to a paired *t*-test (*p* < 0.05). **Bold** indicates optimal performance. ↑: Higher values indicate better performance.

Method	LCS ↑	AUC ↑	F1 ↑	Precision ↑	ECS ↑
FinBERT	0.612	0.701	0.662	0.648	0.645
RoBERTa + MLP	0.638	0.723	0.681	0.667	0.661
XGBoost	0.594	0.689	0.648	0.635	0.602
LSTM	0.617	0.742	0.703	0.689	0.673
Rule-Based Consistency	0.581	0.655	0.590	0.612	0.701
Isolation Forest	0.603	0.731	0.667	0.651	0.612
Multimodal Transformer	0.681	0.785	0.751	0.736	0.742
Contrastive Multimodal Model	0.697	0.803	0.772	0.758	0.768
**Ours**	**0.742**	**0.846**	**0.811**	**0.802**	**0.821**

**Table 4:** Generalization performance under unseen settings. Improvements of the proposed method over the strongest baseline are statistically significant according to a paired *t*-test (*p* < 0.05). **Bold** indicates optimal performance. ↑: Higher values indicate better performance.

Method	LCS ↑	AUC ↑	F1 ↑	Precision ↑	ECS ↑
Text-only Transformer Encoder	0.615	0.703	0.671	0.658	0.640
Behavior-only Temporal Encoder	0.602	0.688	0.654	0.641	0.618
Simple Feature Concatenation (MLP)	0.628	0.721	0.689	0.676	0.662
Attention-based Late Fusion Model	0.651	0.748	0.721	0.707	0.703
Cross-modal Gating Network	0.663	0.762	0.735	0.722	0.718
Dual-stream Contrastive Alignment Model	0.679	0.781	0.754	0.741	0.756
Semantic-Structural Matching Network	0.694	0.803	0.776	0.762	0.781
**Ours**	**0.724**	**0.829**	**0.798**	**0.786**	**0.812**

**Table 5:** Ablation study of proposed framework. Improvements of the proposed method over the strongest baseline are statistically significant according to a paired *t*-test (*p* < 0.05). **Bold** indicates optimal performance. ↑: Higher values indicate better performance.

Variant	LCS ↑	AUC ↑	F1 ↑	Precision ↑	ECS ↑
Full Model	**0.742**	**0.846**	**0.811**	**0.802**	**0.821**
w/o Text Logic Module	0.683	0.791	0.742	0.731	0.742
w/o Behavioral Logic Module	0.671	0.774	0.728	0.712	0.728
w/o Consistency Module	0.645	0.752	0.701	0.688	0.701
w/o Contrastive Learning	0.712	0.818	0.796	0.781	0.804
w/o Logic Regularization	0.728	0.834	0.804	0.792	0.812

## Data Availability

The data presented in this study are available upon request from the corresponding author.
